# Spin-orbital excitations encoding the magnetic phase transition in the van der Waals antiferromagnet FePS_3_

**DOI:** 10.1038/s41535-025-00777-0

**Published:** 2025-06-17

**Authors:** Yuan Wei, Yi Tseng, Hebatalla Elnaggar, Wenliang Zhang, Teguh Citra Asmara, Eugenio Paris, Gabriele Domaine, Vladimir N. Strocov, Luc Testa, Virgile Favre, Mario Di Luca, Mitali Banerjee, Andrew R. Wildes, Frank M. F. de Groot, Henrik M. Rønnow, Thorsten Schmitt

**Affiliations:** 1 PSI Center for Photon Science, Paul Scherrer Institut, CH-5232 Villigen PSI, Switzerland; 2 Laboratory for Quantum Magnetism, Institute of Physics, École Polytechnique Fédérale de Lausanne, CH-1015 Lausanne, Switzerland; 3 Institut de Minéralogie, de Physique des Matériaux et de Cosmochimi, Sorbonne Université, CNRS UMR 7590, 4 Place Jussieu, 75005 Paris, France; 4 Laboratory of Quantum Physics, Topology and Correlations, Institute of Physics, École Polytechnique Fédérale de Lausanne, CH-1015 Lausanne, Switzerland; 5 Institut Laue-Langevin, 71 Avenue des Martyrs CS 20156, 38042 Grenoble Cedex 9, Grenoble, France; 6 Debye Institute for Nanomaterials Science, Utrecht University, 3584 CG Utrecht, Netherlands; 7 Present Address: Deutsches Elektronen-Synchrotron DESY, Notkestraße 85, 22607 Hamburg, Germany

**Keywords:** Magnetic properties and materials, Electronic properties and materials

## Abstract

Van der Waals (vdW) materials are featuring intertwined electronic order and collective phenomena. Elucidating the dynamics of the elementary excitations within the fundamental electronic degrees of freedom is of paramount importance. Here we performed resonant inelastic X-ray scattering (RIXS) to elaborate the spin-orbital excitations of the vdW antiferromagnet FePS_3_ and their role for magnetism. We observed the spectral enhancement of spin-orbital multiplet excitations at about ~100 and ~220 meV, as well as the quasielastic response, when entering the antiferromagnetic phase with an order-parameter-like evolution in temperature. By comparing with model calculations, we discovered the trigonal lattice distortion, spin-orbit interaction and metal-ligand charge-transfer to be essential for these emergent excitations. We further reveal their spectral robustness down to the few atomic-layer limit by mechanical exfoliation, in accordance with the persistent antiferromagnetism reported previously. Our study highlights the crucial role of lattice and orbital anisotropy for stabilizing the quasi-two-dimensional magnetism and tailoring vdW magnets.

## Introduction

Magnetic van der Waals (vdW) materials have provided exciting new opportunities in the studies of functional exotic magnetic phases of various symmetry-breaking ground states and collective behavior^1–4^. Understanding and controlling the spin state and the magnetic exchange interactions are central to establish the next-generation spin-based opto-electronics based on magnetic vdW materials. Recent studies with both optical and X-ray spectroscopy have been utilized to assess the spectral response of the magnetic and electronic configuration down to the few-layer limit^5–8^. These studies resolved the magneto-optical response of the lattice vibrations, and the electronic valence states that give information on spin and orbital configurations, respectively^5,6,8^. However, severe challenges remain for fully understanding the microscopic electronic energy scales in magnetic vdW materials down to the exfoliated few-layer limit. Particularly, many of the exotic spin phases that are of particular interest in realizing spin-based devices occur in the atomically thin two-dimensional (2D) limit, or in the presence of interlayer translational and angular misfit in vdW heterostructures^4,9^. Furthermore, dimensional reduction has been shown to host strong electronic correlations. Advanced spectroscopic techniques with sensitivity to the elementary excitations and the electronic order parameter from the bulk to the few-layer limit of vdW materials are of urgent demand.

Here we present a temperature dependent study of the elementary excitations of the vdW antiferromagnetic insulator FePS_3_ using resonant inelastic X-ray scattering (RIXS). FePS_3_ crystallizes in a honeycomb lattice exhibiting a planar zig-zag antiferromagnetic order with out-of-plane moment orientation below 117 K and anisotropic change of the in-plane lattice constants^10,11^. Raman studies revealed magneto-optical excitations and phonon modes that signified the magnetic phase transition down to the monolayer limit^5,6^. This suggests FePS_3_ as a robust layered nanostructure building block for vdW spintronics. An important outstanding question regards the underlying electronic and spin/orbital structure that enables quasi-2D antiferromagnetism down to the few-layer limit. Such information will guide future research on pathways to tune the low-energy instabilities in FePS_3_, which may provide crucial insight for device applications.

The central question here is: how is the long-range magnetic order in a quasi-two-dimensional lattice environment stabilized? Such long-range order generally requires additional anisotropic interactions as governed by the Mermin-Wagner theorem^12^. In low-dimensional spin systems, the natural candidates that introduce anisotropy and symmetry-breaking are the magneto-crystalline interactions. Specifically, the zig-zag magnetic order and corresponding local structural nuances are expected to lead to spin-lattice coupling^10^. Pump-probe optical studies reported the ultrafast control of antiferromagnetic order in FePS_3_, and revealed that the orbitals’ symmetries can be described by a trigonal distortion of the local FeS_6_ cluster^13^ with signatures of phonon-magnon coupling^14^. However, Fe *L*_3_-edge X-ray absorption spectroscopy (XAS) experiments have remained inconclusive on whether the local C_3_ lattice symmetry-breaking plays a significant role for the emergent magnetic zig-zag orders^8,15^. Despite the existing studies that quantify the local site nuances beyond cubic-symmetry octahedral environments^16,17^, the exact electronic ground state can be sensitive to the trigonal distortion and how it couples to magnetism, which has not been quantified in a collective effort for FePS_3_.

To resolve these issues, we use Fe *L*_3_-edge RIXS to study the low-energy excitations of FePS_3_. RIXS is a spectroscopic technique that probes the dynamics of the elementary excitations in condensed matter^18,19^. The two-step scattering processes of RIXS grant access to a variety of charge-neutral excitations. Transition-metal *L*-edge RIXS studies have revealed optically-forbidden modes such as single magnon and multiplet excitations with spin-orbital characteristics^20^, and were recently extended to studies investigating few atomic-layer samples^7,21–23^. Particularly, the low-energy spin-orbital excitations have provided valuable information on the low-energy physics of Fe-based correlated materials using RIXS, with strong temperature and polarization dependence due to the presence of the ordered spin and orbital texture^24,25^. In this work, we report the temperature dependence of the elementary excitations in bulk single crystals of FePS_3_ using Fe *L*_3_-edge RIXS. Around the Fe *L*_3_-edge XAS peak maxima (E_*i*_ = 706.5 and 707.5 eV), we observe two distinguishable modes at about ~100 and ~220 meV that clearly evolve with the magnetic state of the material. These predominant spectral peaks clearly suppress upon heating above the Néel temperature, with a crossover-like behavior for the observed excitations. By comparison to ligand-field calculations with charge-transfer configuration interaction, we find that the ground state of Fe in FePS_3_ originates from a ^5^E_1_ symmetry. The first low-energy spin-orbital excitation at ~100 meV can be assigned to excitations to the ^5^E_1_ manifold that is split by spin-orbit interaction and magnetic exchange interaction. The second low-energy spin-orbital excitation at ~220 meV can be assigned to excitations to the ^5^A_1_ and ^1^A_1_ manifolds, which is split by the magnetic exchange interaction. Furthermore, the energy position of the ~220 meV feature is directly determined by the magnitude of the trigonal distortion providing us with a robust quantification of the distortion.

Importantly, we find that the trigonal C_3_ distortion, spin-orbit coupling, and metal-ligand charge-transfer are mandatory to reproduce the observed doublet-peak profile with physically reasonable electronic parameters, as well as their temperature development across the magnetic phase transition. Lastly, spectral comparison of bulk with exfoliated few-layer samples reveals the robustness of the electronic structure down to 5 monolayers (MLs), giving an explanation of the reported persisting antiferromagnetism in few-layer samples. Our findings provide a benchmark for RIXS studies in vdW materials hosting magnetic order in general, and shed light on the exotic spin-orbital ground/excited state properties of FePS_3_.

## Results

### Overview of the experimental RIXS results

**Fig. 1:**
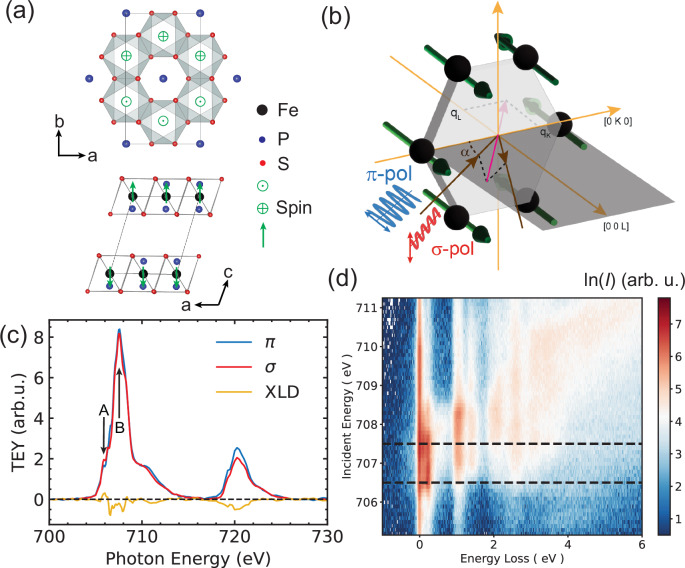
Crystal structure, magnetic order, XAS and energy-dependent RIXS overview, and scattering geometry. **a** Geometry of the Fe honeycomb plane and interlayer antiferromagnetic structure visualized by the authors using VESTA^60^, where green arrows show the direction of spin. **b** Schematics for experimental geometry created by the authors using Abode Illustrator software package. XAS and RIXS spectra were measured at an incident angle *α* = 12.5°. **c** XAS in total electron yield (TEY) of FePS_3_ measured at 200 K, with the corresponding X-ray linear dichroism (XLD). A = 706.5 eV and B = 707.5 eV mark the two main incident energies of interest as also highlighted in the RIXS map in (**b**) (black dashed horizontal lines). **d** RIXS map of FePS_3_ as a function of incident photon energy (left axis) and energy loss (bottom axis). *π* polarization is employed in this measurement. The RIXS intensity is plotted in logarithmic scale. This figure does not contain any element that requires copyright permission, but only experimental data that the authors measured or self-created schematic graphics that were not taken from other sources.

In FePS_3_, the Fe atoms are arranged in a honeycomb lattice with stacked layers as shown in Fig. 1a. The magnetic structure has been confirmed to have a collinear antiferromagnetic structure with the moments normal to the ab plane^10^, also shown in Fig. 1a. The Fe *L*_3_-edge XAS spectra are shown in Fig. 1c, which are in agreement with recent works^8,15^. The overall XAS spectral shape indicates that Fe ions participate in a partially ionic bond as evidenced by the sharp multiplet features in the energy range between 700 and 709 eV. In addition, spectral signatures of strong hybridization in the bonds between Fe and S are also evident where there is a strong charge transfer peak observed at the energy range between 709 and 714 eV. This agrees well with literature on sulphide compounds^26,27^ and is confirmed by our calculation where we found that the XAS spectra can be well reproduced by d^6^ + d^7^*L* electronic configuration of the Fe^2+^ ions, where *L* denotes the ligand hole state. (see Supplementary Fig. 6) We conclude that the electronic structure of FePS_3_ is best described with *a* | *d*^6^ > + *b* | d^7^*L* > wavefunction, where |*a*|^2^ ≃ 68.5% and |*b*|^2^ ≃ 31.5%, in order to reproduce the Fe *L*_3,2_-edge absorption profile (see later subsection “Calculated electronic and spin-orbital states in the RIXS spectra”). We present RIXS measurements in the experimental geometry depicted in Fig. 1b as a function of incident photon energies E_*i*_ across the Fe *L*_3_-edge XAS resonances at the base-temperature 20 K in Fig. 1d (see same measurements above the antiferromagnetic transition in Supplementary Fig. 2). We observed a series of localized Raman-like excitations up to 3 eV and fluorescence-like excitations up to about 5 eV energy loss. In a simplified high-spin octahedral crystal-field environment, the Fe^2+^ electronic ground state is formed from a ^5^D group (*L* = 2) atomic multiplet, with the ^5^T_2*g*_ ground state and first excited state of ^5^E_*g*_ symmetry in the weak crystal field limit^28,29^. Indeed, the localized peaks about 1 and 1.3 eV loss resemble the spin-allowed ^5^T_2*g*_ to ^5^E_*g*_ transitions^28,29^, with a suggested broadening for the 1 eV peak from the dynamical Jahn-Teller effect^30^. In the higher-energy regime of 3-5 eV loss, we observe a fluorescence-like spectral response that is resonant about the post-edge broad band in XAS (E_*i*_ = 710 eV). These are reminiscent of the charge-transfer processes that were previously attributed to transitions from the S 3p_*x*_p_*y*_ orbitals to the empty Fe 3d shell, as well as the transitions from P-P 3p_*z*_ to S 3${{\rm{p}}}_{z}^{* }$ states^31^. At lower-energy loss shown in Fig. 1d, we observed a peculiar spectral characteristics below 500 meV. These inelastic modes of near-infrared energy range were not covered in previous theoretical or experimental studies. To understand their nature and interplay with magnetism, we performed further temperature dependent RIXS experiments.

### Temperature dependence of the experimental RIXS results

**Fig. 2:**
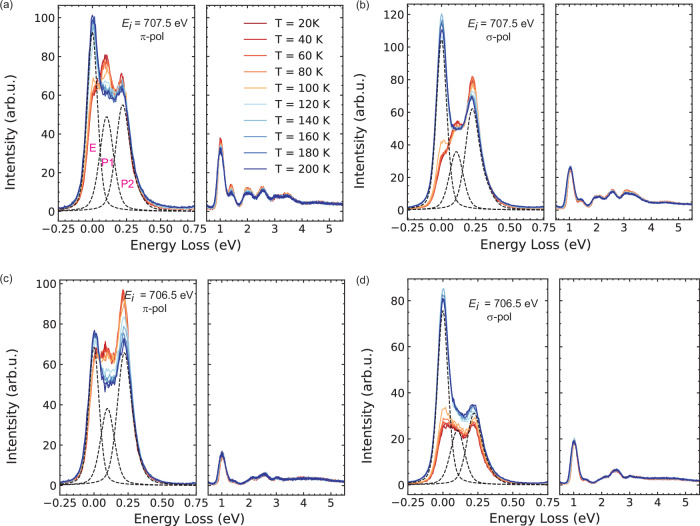
Temperature-dependent RIXS spectra. RIXS energy transfer spectra at different temperature with 707.5 and 706.5 eV incident energy in (**a**, **b**) and (**c**, **d**); spectra recorded for *σ* and *π* incident X-ray polarization are shown in (**a**, **b**) and (**c**, **d**), respectively. Each graph has panels for the low-energy excitations below 0.5 eV including the elastic peak E and spin-orbital peaks P1/P2 (left) and the high-energy excitations up to 4.5 eV (right). All data was obtained at an experimental geometry with 12.5° incidence angle.

In Fig. 2, we show the temperature evolution of RIXS spectra taken at E_*i*_ of 706.5 and 707.5 eV with both *π* and *σ* polarization for the incident X-rays. These two resonant energies correspond to the Fe *L*_3_-edge XAS maximum B and a pre-edge shoulder peak (A in Fig. 1c), where the predominant low-energy excitations below 500 meV loss are enhanced in spectral intensities (see RIXS spectra taken at other resonances in Supplementary Fig. 3). A zoom into this low-energy regime is highlighted in panels separated from panels of the higher-energy range above 1 eV loss. We observed two distinguishable energy loss peaks centering around ~100 meV and ~220 meV energy loss. Here we designate the ~100 and ~220 meV excitations as peak 1 and 2, respectively (see fitting assignment in Supplementary Fig. 4). These excitations are multiplet excitations due to the fine structure of crystal field splitting, spin-orbit coupling effects and magnetic exchange interaction, termed spin-orbital excitations as in Fe *L*_3_-edge RIXS studies on magnetite Fe_3_O_4_^24,25^. With increasing temperature, we found that peak 1 exhibits clear suppression at both E_*i*_ of 706.5 and 707.5 eV with *π* polarization. This phenomenon becomes more drastic in vicinity to the magnetic phase transition. While the same evolution can be observed for peak 2 at E_*i*_ of 706.5 eV with *π* polarization, it becomes nearly temperature-independent for E_*i*_ = 707.5 eV. On the other hand, both peak 1 and 2 show less temperature development with *σ* polarization. We note that the quasi-elastic peak E undergoes a steep rise right after entering the high-temperature paramagnetic phase upon heating above Néel ~120 K, which is stronger with *σ* polarization. Lastly, excitations above 1 eV energy loss exhibit weak temperature developments in general.

These findings resemble the fine structure of active t_2*g*_ orbital energy levels, which are sensitive to exchange fields, spin-orbit coupling and lattice distortions^25,32–34^. The latter naturally connects to the discontinuous decrease (increase) of magnitude in lattice constant **a** (**b**) at the magnetic transition^35^, suggesting a possible magneto-restriction mechanism for the observed temperature developments of the low-energy excitations peak 1 and 2. Such strong spin-lattice coupling is evidenced by the previous high-field magnetization experiments and anisotropic exchange model calculations^36,37^. The incidence angle dependence with light polarization follows previous studies on multiplet transitions of a FeS_6_ cluster configuration (see Supplementary Fig. 5). Lastly, the ~220 meV peak 2 is also broadly consistent with the observed spectral profile in a former neutron study^38^. On the other hand, the differences in the details of the temperature dependence (independence) for peak 2 taken at E_*i*_ = 706.5 (707.5) eV shown in Fig. 2a, b might relate to the different intermediate and final states involved in the RIXS processes^18^. As for the elastic peak E enhancement upon heating, this phenomenon resembles the increasing weight for the spin-singlet state in other reports^39,40^.

### Calculated fitted electronic and spin-orbital states in the RIXS spectra

**Fig. 3:**
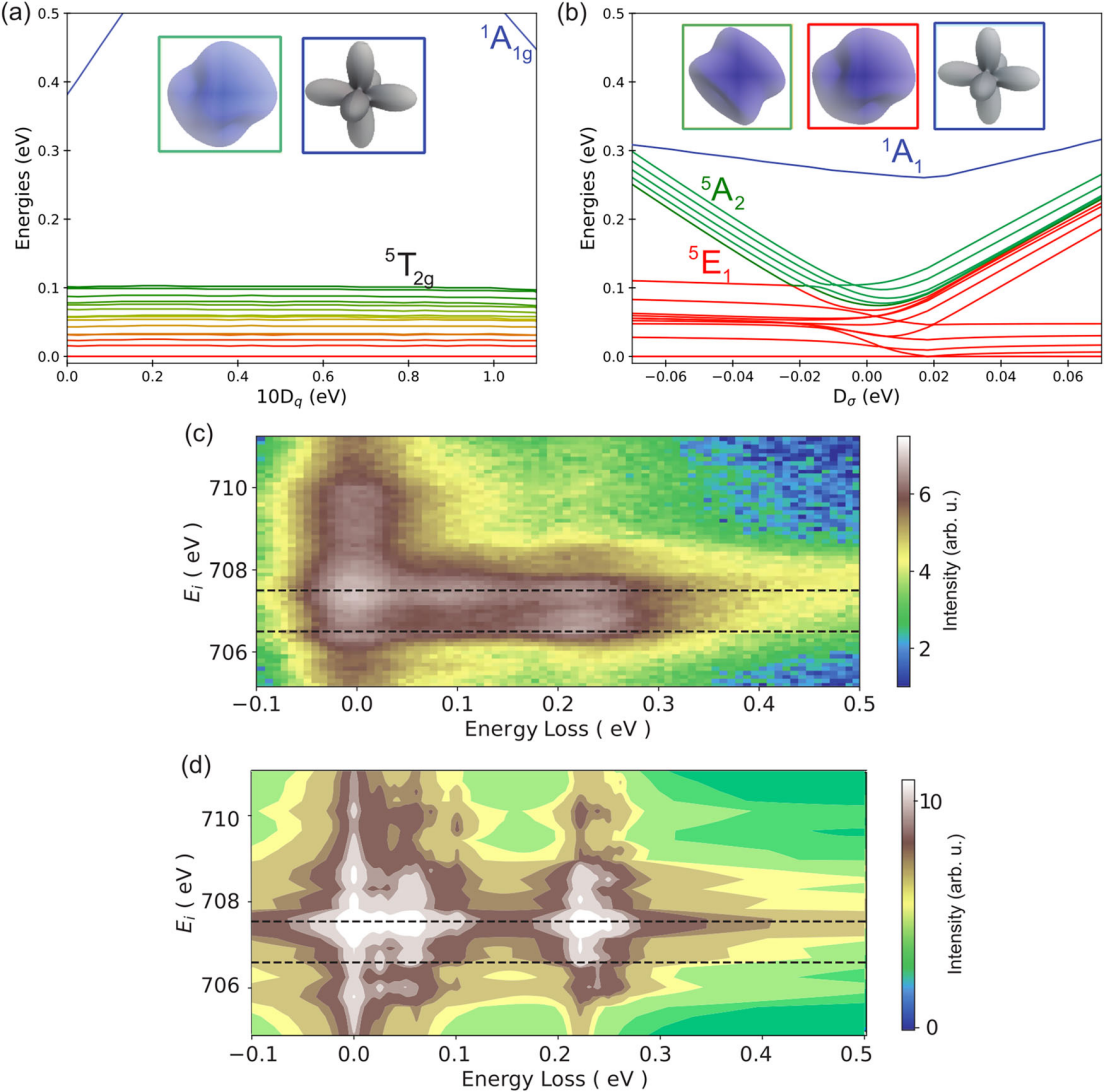
Multiplet ligand field theory calculations. Energy level diagram of multiplet states for Fe^2+^ ions in FePS_3_ together with the RIXS spectral response determining the distortion parameters. **a** The effect of 10*D*_*q*_ parameter representing the octahedral crystal field varied from zero to 1 eV. **b** The effect of *D*_*σ*_ parameter representing the magnitude of trigonal distortion. Representative charge density plots of the multiplets are shown above color coded to the states. The color of the density plots represents the spin projection where blue is down, gray is 0 and red is up. **c** An experimental RIXS map with incident photon energy in *σ* polarization. **d** Full RIXS map calculations around the Fe *L*_3_-edge. A set of distortion values of -60 and 0 meV for D_*σ*_ and D_*τ*_, respectively, were employed. The multiplet parameters were optimized to quantitatively describe the experimental data after experimental resolution broadening. The calculated intensities are averaged over *π* and *σ* polarization for the outgoing X-rays.

To understand our RIXS inelastic response, we performed charge-transfer multiplet (CTM) theory calculations to determine the electronic structure of FePS_3_ and pinpoint the interactions leading to peak 1 and 2. To begin with, the ground state of a high-spin Fe^2+^ ion (3d^6^) in cubic symmetry is ^5^T_2*g*_, which is composed of 15 micro-states that are split due to magnetic exchange interaction and spin-orbit coupling. The energy level diagram of Fe^2+^ taking into consideration three charge transfer configurations, i.e. d^6^ + *d*^7^*L* + *d*^8^*L*^2^ shown as a function of the octahedral crystal field parameter (10*D*_*q*_) in Fig. 3a (*d*^8^*L*^2^ term was confirmed to be significantly smaller than the *d*^7^*L* contribution, which agrees with former studies comparing 3d^6^ high- and low-spin transition-metal compounds^41^). It is expected that the octahedral crystal field is ~1 eV for TMPS_3_ compounds (TM = Mn, Fe, Co, Ni) leading to a high spin-ground state^42^. This leads to a situation where the 15 states belonging to ^5^T_2*g*_ span the energy range 0–100 meV, followed by the ^1^A_1*g*_ state which shows a strong dispersion as a function of 10*D*_*q*_. It is important to note here that the lowered energy position of the ^1^A_1*g*_ state is a direct consequence of the strong hybridization between Fe and S. More details about the effect of charge-transfer can be found in the Supplementary Fig. 8. Density plots of representative wave functions belonging to the ^5^T_2*g*_ (in red box) and ^1^A_1*g*_ (in blue box) multiplets are shown at the top of Fig. 3a.

It is clear that an additional distortion is necessary to obtain the second RIXS feature at ~ 220 meV. We investigated the effect of a trigonal distortion on the FeS_6_ local octahedral environment^13^. Figure 3b shows the effect of the trigonal distortion parameter, *D*_*σ*_ on the energy diagram. This is a new aspect that was not observed for NiPS_3_^42^, yet it agrees with most of the literature on FePS_3_^29^ including the very recent results of X-ray photoemission electron microscopy on FePS_3_^8^. The trigonal distortion splits the ^5^T_2*g*_ multiplet to ^5^E_1_ (10 states) and ^5^A_2_ (5 states) as shown in Fig. 3b. Here we found that the distortion parameters *D*_*σ*_ and *D*_*τ*_ of -60 and 0 meV, respectively, with crystal-field 10*D*_*q*_ = 1 eV can explain our experimental data trend. This means that peak 1 at ~100 meV corresponds to an excitation in to the higher-lying states of the ^5^E_1_ multiplet. The energy splitting between the 10-fold ^5^E_1_ states is determined by the magnitudes of the spin-orbit coupling and the magnetic exchange interaction (see Supplementary Fig. 9). On the other hand, peak 2 at ~ 220 meV corresponds to an excitation mainly to the ^5^A_2_ multiplet for which its energy position is directly determined by the magnitude of the distortion parameter *D*_*σ*_ and its splitting within the multiplet depends only on the exchange interaction. A small shoulder can be observed at the high energy end of peak 2 which corresponds to transitions to the ^1^A_1_ state. Our quantification of the magnitude of the distortion parameter is particularly robust, as seen in Fig. 3b due to the direct relation of the energy position of peak 2 on the magnitude of the distortion *D*_*σ*_. A comparison between the experimental and theoretical RIXS map is shown in Fig. 3c, d, and reproduces the two low-energy peaks 1 and 2. We conclude therefore that the presence of a local trigonal distortion, spin-orbit coupling and charge-transfer is essential to reproduce our experimental observation. An interesting aspect of our solution is the proximity of the ^1^A_1_ state to the ground state with only 300 meV of difference due to the strong hybridization between Fe and S. As the ^1^A_1_ state strongly disperses as a function of distortion, it becomes the ground state at *D*_*τ*_ = 50 meV (also see Supplementary Figs. 7–8).

**Fig. 4:**
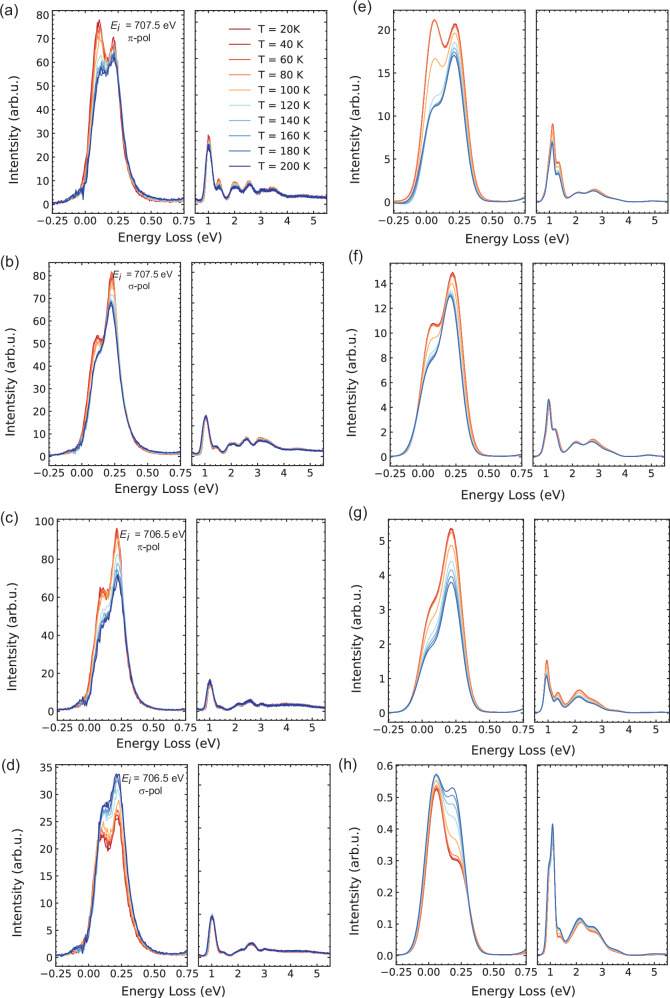
Spectral comparison between experimental and theoretical RIXS response with temperature. Experimental and calculated spectra with elastic line subtracted at different temperature, represented by different colors as denoted in (**a**). **a**–**d** show the experimental results, while (**e**–**h**) present the corresponding theoretical calculations.

**Fig. 5:**
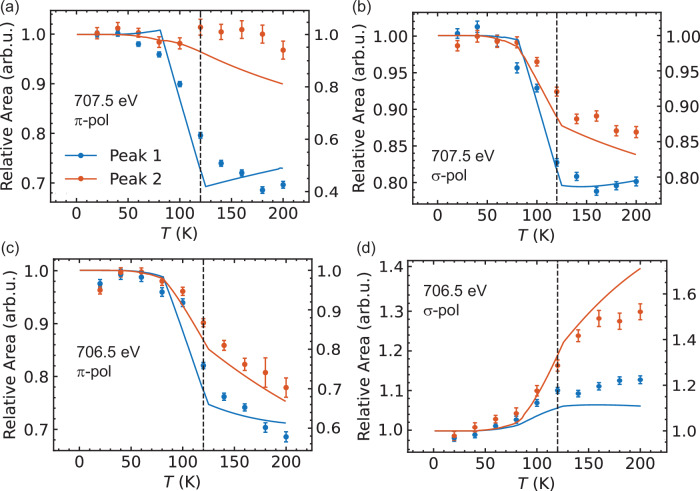
Fitted intensities comparing experimental and theoretical RIXS response with temperature. Temperature dependence of the RIXS intensity of the spin-orbital excitations across the magnetic phase transition temperature. **a**–**d** Results for different incident energies and polarizations, as indicated in the legend. All intensity values are represented relative to the value at 20 K for comparative analysis. The experimental fitting spectra weights are shown as dots, while the calculation results are represented by solid lines, with intensity scales on the left and right axes, respectively. The antiferromagnetic ordering temperature ~120 K is indicated by a black dashed line in all panels. The errors are standard deviations of the fitting results.

We then compare the temperature evolution of the experimental RIXS response and the calculated spin-orbital excitations. Full spectral comparison and the respective fitted peak weight contribution are shown in Figs. 4 and 5, respectively. The elastic line contribution was subtracted from both the experimental and calculated spectra. The overall lineshapes in the experimental and theoretical spectra exhibit similar trends, indicating that the key spectral features are well captured by the model simulations. In these calculations, we include the magnetic exchange interaction between different Fe sites on a mean-field Heisenberg level. The mean field magnetic exchange interaction for site i is: H_*exch,i*_ = J∑_*j*_ < S_*j*_ > ·S_*i*_ where j are the neighbor equivalent Fe sites. Similarly for site j: H_*exch,j*_ = J∑_*i*_ < S_*i*_ > ·S_*j*_. We solved these equations self-consistently including the full Hamiltonian (i.e. crystal field, electron-electron repulsion and spin-orbit coupling) at each temperature. To verify our model, we computed the temperature behavior of the magnetic susceptibility which agrees reasonably well with experimental data (see Supplementary Fig. 11). Our theoretical model captures the main essence of our experimental observations: (1) The intensities of the spin-orbital excitations are strongly modified at the magnetic transition temperature, exhibiting a two-state like crossover. (2) The order of magnitude in intensity changes and effects of light polarization are captured. The remaining differences, including some intensity background sources that gradually change with temperature without a steep trend at the magnetic phase transition, may be attributed to additional factors like Boltzmann population broadening via phonon coupling or short-range spin fluctuations. Neutron studies have shown a Lorentzian-like quasi-elastic weight extending to ~40 meV and persisting above the antiferromagnetic transition, suggesting possible non-negligible short-range spin fluctuations in the paramagnetic phase^43^ which can not be captured by a simple mean field model (also see Supplementary Fig. 12). Nevertheless, our experimental RIXS response can be reasonably captured by CTM theory with a two-state crossover across the magnetic phase transition, signifying an order-parameter-like development.

We remark that we do not focus on the temperature evolution of the quasi-elastic peak E despite it showing a pronounced step like temperature development when passing through the Néel transition temperature of ~120 K as illustrated in Supplementary Fig. 10. Its intensity is affected by several factors in our experiments, such as the non-resonant elastic X-ray scattering, low-energy resolution-limited excitations (e.g. magnons^10^, phonons^5^, multiplet excitations^39,40^, etc), diffuse scattering due to sample surface irregularities, self-absorption and saturation effects, etc. These factors are not included in our simulations, and hence it is very difficult to understand the temperature dependence of the quasi-elastic and disentangle these contributions. However, we propose that the strong temperature dependence of the quasi-elastic peak E may be linked to proximity of the ^1^A_1_/^5^E_2_ states, which can possibly be enabled by a small (dynamical) distortion switching the order of the states. In this picture, when entering the paramagnetic phase, the restored degeneracy for the lowest-lying ^1^A_1_/^5^E_2_ ground states may lead to an enhanced spectral weight at zero-energy loss (see Supplementary Section 5.2). It was rationalized that the high-temperature paramagnetic phase could favor such raised symmetry for s-wave like spin and orbital states^39,40^. This scenario, however, would require further investigations with significantly higher energy resolution.

### Exfoliated thin flake RIXS response

**Fig. 6:**
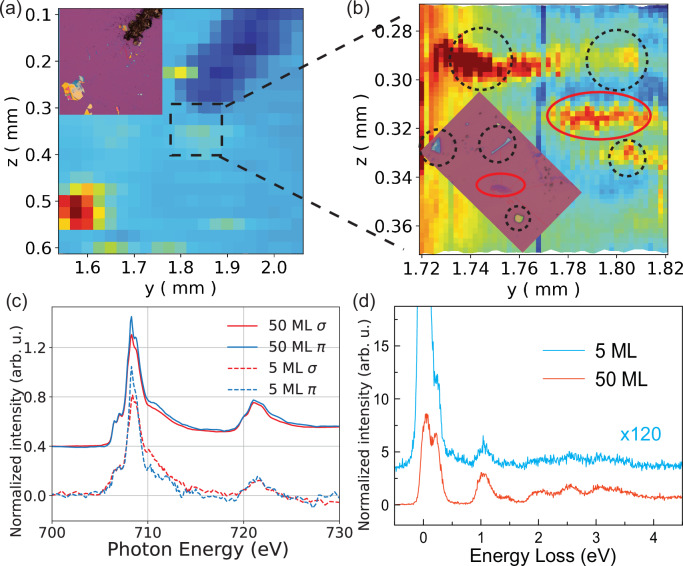
Exfoliated thin flake XAS and RIXS response. **a** The optical microscopy image (insert top left) and spatial map of total fluorescence yield at Fe *L*_3_-edge absorption maximum for position registry of the mechanically exfoliated FePS_3_ flake samples with 5 ML thickness on a SiO_2_/Si substrate. **b** Zoom-in region around the measured flakes with the corresponding optical image and XAS spatial map. The 5 ML sample and the surrounding thicker bulk-like flakes, including the thickest near the top left corner (50 ML), are highlighted by the red solid-line ellipse and black dotted-line circles, respectively. **c**, **d** XAS and RIXS data recorded on exfoliated 50 ML and 5 ML samples at 20 K. RIXS data for 5 ML sample is scaled up in spectral intensity for clarity in comparison.

Lastly, we assess the response of these spin-orbital excitations towards the 2D limit by employing RIXS measurements in mechanically exfoliated samples. In Fig. 6a, b, optical microscopy images and XAS spatial mapping of the exfoliated samples are shown and cross-compared for position registry. The sample flake preparation and thickness characterization can be found in Supplementary Section 8 and Supplementary Fig. 13. The corresponding XAS and RIXS spectra are shown in Fig. 6c, d. We observe that the overall XAS spectra and RIXS profiles extending to higher-energy excitations persist in the exfoliated flakes of 50 atomic layers (50 ML) and atomically thin 5 MLs thickness. In general, our results suggest the absence of large chemical changes or other variation in these samples at the ultra-thin limit. This shows that the multiplet excitation response, and thus the electronic structure, of the bulk phase remains intact in the exfoliated thin flakes. The low-energy peak 1 of the bulk phase (~100 meV) is more difficult to assess in few-layer samples of 5 MLs due to the substrate diffuse (elastic) scattering and the correspondingly lower signal level. Nevertheless, the persistence of the multiplet excitation response may contribute to the explanation of the robust antiferromagnetism in FePS_3_ down to the few-layer limit as observed in previous optical measurements^6^.

## Discussion

We demonstrate the versatile RIXS sensitivity to low-energy excitations in vdW antiferromagnet FePS_3_ from spin-orbital multiplet transitions that grant access to the microscopic electronic interactions. The temperature- and polarization-resolved RIXS measurements shows an evolution of the low-energy dynamics at ~100 meV and ~220 meV that correlates with the development of the magnetic order parameter and the underlying electronic wavefunction. From the observation of these spin-orbital excitations registering the magnetic phase transition, our study allows the assessment of the crucial role of trigonal local distortions and the metal-ligand charge transfer. With the aid of ligand-field theory calculations, we capture the essential electronic interactions that stabilize the long-range antiferromagnetism in the quasi-2D limit, which we find to persist in our RIXS and XAS measurements comparing the bulk crystals and exfoliated 5 ML flake samples. Recently, RIXS has been shown as a powerful spectroscopic tool to investigate electronic structure and dynamics at distinct phases as a function of temperature^44–48^. On this front, our major achievement constitutes the investigation of the temperature evolution of spin-orbital multiplet excitations probed by the RIXS cross-section, which directly reveal an order-parameter-like development of the dynamical spectral weight across the magnetic phase transition. The control of these ground state properties is a prerequisite for material engineering utilizing spin-lattice interactions and charge-transfer energetics, e.g. piezo-control electronics with strain tuning, spin-flip photoluminescence sensors in OLEDs and quantum sensing^49^. Our work highlights RIXS as an ideal investigation tool for studying the electronic structure of functional 2D materials, featuring sensitivities to all electronic degrees of freedom under flexible parameter control.

## Methods

### Resonant inelastic X-ray scattering experiment

Fe *L*_3_-edge resonant inelastic X-ray scattering (RIXS) and X-ray absorption spectroscopy (XAS) experiments were performed at the Advanced Resonant Spectroscopies (ADRESS) beamline of the Swiss Light Source at the Paul Scherrer Institut^50–52^. The total energy resolution of the RIXS experiment was about 85 meV at the Fe *L*_3_-edge (≈707 eV). The RIXS spectrometer was fixed at a scattering angle 2*θ* = 130°. An experimental geometry was used that was fixing the scattering plane in the *bc* plane with the in-plane momentum transfer along the crystallographic [010] direction, with an incidence angle of 12.5°. The corresponding momentum transfer vector **q** is [0, 0.844, 0.394], based on the space group *C*2/*m*, with lattice parameters *a* = 5.94(4) Å, *b* = 10.26(2)Å, *c* = 6.60(6) Å, and *β* = 108.3(7) Å^10^. RIXS spectra were acquired with 15 (1) minutes for the temperature (incident energy) dependent measurements, respectively, and normalized to the incoming beamline flux. XAS spectra were recorded in total electron yield (TEY) and total fluorescence yield (TFY) mode. Both *π* and *σ* polarization was employed for the incident X-rays. Unless specified, all RIXS and XAS measurements were performed at the base temperature at 20 K. Details on the synthesis of single crystal FePS_3_ samples can be found elsewhere^10^.

### Multiplet ligand field theory calculations

To understand the character of the low-energy excitations peak 1 and 2, we employ exact diagonalization calculations within charge-transfer multiplet (CTM) theory as implemented in Quanty^53–55^. The model reduces to a multi-electronic calculation of a single FeS_6_ cluster, accounting for the Fe-3*d* orbitals and the corresponding symmetrized molecular orbitals from Fe 3d states with hybridization to S 3p states^53,56,57^. The cluster has a trigonal-symmetry which is quantified through three distortion parameters (D_*q*_, D_*σ*_ and D_*τ*_). Configuration interaction calculations taking into account (i) the intra-atomic Coulomb interaction, (ii) the crystal field, (iii) charge transfer, (iv) the mean field exchange interactions, and (v) spin-orbit interaction were performed using the quantum many-body program Quanty^53–55^. Briefly, the ligand-field interaction is given by three different terms. These are the on-site splitting on the transition-metal d-shell, the on-site splitting on the ligand p-shell, and the hopping between the ligand p-shell and the transition-metal d-shell. The ligand states in consideration are made of linear combinations of interacting S-3p orbitals. This reduces the 36 S-3p orbitals only to 10 interacting orbitals with the Fe-d orbitals. The on-site energies can be related to the charge-transfer energy (Δ), and the d-d Coulomb interaction (U) which includes exchange interactions following the definitions by the original work of Zaanen, Sawatzky and Allen^58^. The energy differences are referred from the center of the ligands and transition metal sites after the application of the d-electron crystal field. The following equations define the relation between U_*dd*_ (the on-site Coulomb repulsion between two electrons on the metal d orbitals), Δ (the charge transfer energy), and the onsite energies of the ligand (*ϵ*_L_) and d orbitals (*ϵ*_*d*_) with n being the formal valency (*n* = 6 for Fe^2+^):

$${\epsilon }_{d}=\frac{\left(10* \Delta -n* \left(19+n\right)* {U}_{{dd}}/2\right)}{\left(10+n\right)}$$

$${\epsilon }_{L}=\frac{n* \left(\left(1+n\right)* {U}_{{dd}}/2-\Delta \right)}{\left(10+n\right)}$$

The value of mean field exchange *J*_*exch*_ is adapted from a previous neutron scattering study^10^. The charge transfer and orbital hybridization are kept fixed above and below the antiferromagnetic phase transition, while small perturbations due to changes in surrounding spin environments may be at play and would require future investigations^59^. The *d* − *d* and *p* − *d* multipole part of the Coulomb interaction was scaled to 80% of the Hartree-Fock values of the Slater integral. Three charge transfer configurations were taken into account in the calculations (i.e., 3*d*^*n*^ + 3*d*^*n*+1^*L* + 3*d*^*n*+2^*L*^2^). The parameters used for the multiplet calculations are specified in Table 1.

**Table 1:** Input parameter for multiplet calculations of Fe in FePS_3_

(eV)	10*D*_*q*_	*D*_*σ*_,*D*_*τ*_	Δ	*V*_*a*1_*,V*_*b*1_	*Ve*,*V*_*b*2_	*U*_*pd*_	*U*_*dd*_	*J*_*exch*_	*SOC*_*3d*_
Initial	1	−0.06,0	0.98	1.5	0.7	-	5	0.012	0.052
Intermediate	1	−0.06,0	-0.55	1.5	0.7	6	5	0.012	0.0665

Here the trigonal crystal field parameters are given by 10D_q_, D_σ_ and D_τ_. The charge transfer energy and hybridization are given by Δ and V. The onsite energies are given by U_pd_ and U_dd_. The mean field exchange field is J_exch_ and the spin-orbit coupling constant is referred to as SOC_3d_.

## Supplementary information

supplementary information

## Data Availability

All data needed to evaluate the conclusions are present in the paper and/or the supplementary information. The raw data files will be available upon paper acceptance at a public repository with access link: https://zenodo.org/records/15039939.

## Code availability

All the accession codes used in the present manuscript and supplementary information are available upon reasonable request.
