# Supplementary material for: Spin-orbital excitations encoding the magnetic phase transition in the van der Waals antiferromagnet FePS3
Source: NPJ Quantum Mater. 2025 Jun 17;10(1):61. doi: 10.1038/s41535-025-00777-0 (PMC12173934; doi:10.1038/s41535-025-00777-0)
Supplement: Supplementary file 1 — supplementary information [file 41535_2025_777_MOESM1_ESM.pdf]

Supplementary Information for: Spin-orbital excitations encoding the magnetic phase transition in the van der Waals antiferromagnet $FePS_3$

Yuan Wei[1,*,†], Yi Tseng[1,2,*,‡,†], Hebatalla Elnaggar[3,*], Wenliang Zhang[1], Teguh Citra Asmara[1], Eugenio Paris[1], Gabriele Domaine[1,2], Vladimir N. Strocov[1], Luc Testa[2], Virgile Favre[2], Mario Di Luca[4], Mitali Banerjee[4], Andrew R. Wildes[5], Frank M. F. de Groot[6], Henrik M. Rønnow[2], and Thorsten Schmitt[1,†]

[1]Photon Science Division, Paul Scherrer Institut, Villigen PSI, Switzerland
[2]Laboratory for Quantum Magnetism, Institute of Physics, École Polytechnique Fédérale de Lausanne, CH-1015 Lausanne, Switzerland
[3]Institut de Minéralogie, de Physique des Matériaux et de Cosmochimi, Sorbonne Université, CNRS UMR 7590, 4 Place Jussieu, 75005 Paris, France
[4]Laboratory of Quantum Physics, Topology and Correlations, Institute of Physics, École Polytechnique Fédérale de Lausanne, CH-1015 Lausanne, Switzerland
[5]Institut Laue-Langevin, 71 Avenue des Martyrs CS 20156, 38042 Grenoble Cedex 9, France
[6]Debye Institute for Nanomaterials Science, Utrecht University, 3584 CG Utrecht, Netherlands
[*]these authors contributed equally to this work
[‡]Present address: Deutsches Elektronen-Synchrotron DESY, Notkestraße 85, 22607 Hamburg, Germany
[†]email: yuan.wei@psi.ch; yi.tseng@desy.de; thorsten.schmitt@psi.ch

May 5, 2025

Supplementary Note 1 Extended XAS results

Here we show extended experimental X-ray absorption spectroscopy (XAS) results above and below magnetic phase transition ∼120 K with both total electron yield (TEY) and total fluorescence yield (TFY) channels recorded. This is shown in Supplementary Figure 1. In TFY channel, we observe clear suppression of spectral weight fraction of Fe L_3-edge (peaked ∼708 eV) compared to L_2 (peaked ∼721 eV), which contrasts to the present XAS reports and our main Figure 1 (also see references thereof). This could be accounted for by both spectral saturation effects at the Fe L_3-edge white line, as well as the self-absorption distortion from the outgoing fluorescence photons. Additionally, the temperature-dependent background level in TFY XAS, based on fluorescent detection by a photodiode, could artificially alter the spectral intensity levels, thereby altering the anticipated spectral trend. (e.g. X-ray linear dichroism was suppressed and reversed in polarization orientation in Supplementary Figure 1d) This contrasts to the inelastically scattered photon detection for RIXS. The CCD detector employs an algorithm for photon event centroiding that makes count discretion with intrinsic background subtraction from the count histogram, which is based on the given spatial distribution of photo-excited charge clouds on the CCD. On the other hand, these XAS artifacts are largely improved with the TEY detection. The low-temperature TEY data taken at T = 20 K showed significantly worse signal-to-noise ratio compared to the higher temperature results taken at T = 200 K. This can be understood by the thermally activated electrons across band gap at 200 K while the $FePS_3$ sample remains reasonably insulating at lower temperature T = 20 K. Lastly, both TEY and TFY XAS results show sizable spectral changes across the magnetic phase transition ∼120 K. Nevertheless, their spectral developments and sensitivity to magnetism are obscured by either insufficient statistics (TEY) or saturation/self-absorption effects that can bear a temperature-dependent background. Both cases would require further investigations in the future.

Supplementary Note 2 Additional incident-energy dependent RIXS data

Here we report additional incident-energy dependent resonant inelastic X-ray scattering (RIXS) data. These results are performed in the same way as the measurements shown in main text for the RIXS energy map (Figure 1d) and temperature-dependent RIXS linecuts (Figure 2), respectively. The experimental configuration is fixed at a scattering angle $2\theta = 130°$, with the same bc scattering plane and the in-plane momentum transfer along the crystallographic [010] direction in 12.5° grazing incidence. In Supplementary Figure 2, we present the incident-energy dependence of RIXS spectra taken at 200 K, which is well above the antiferroamgnetic ordering temperature ∼120 K. Except for the uprising intensity of the elastic line centering at zero-energy loss, we observe that

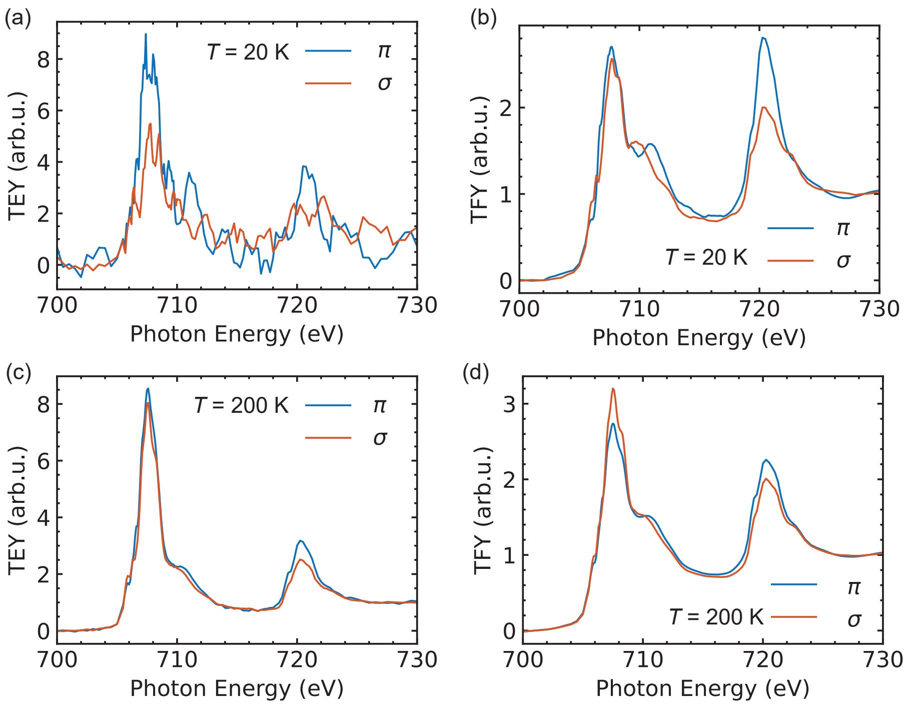

Supplementary Figure 1: Low-temperature (T = 20 K) XAS spectra taken below magnetic phase transition temperature for a, TEY and b, TFY channel, respectively. High-temperature (T = 200 K) XAS spectra taken above magnetic phase transition temperature for c, TEY and d, TFY channel, respectively.

all the inelastic RIXS modes exhibit an overall weight suppression upon heating.

On top of this, we further explore the temperature developments at other selected incident-energies across the Fe L_3-edge XAS profiles. Here we focus on the excitation profiles taken at distinct resonances away from the pre-edge and maximum of Fe L_3-edge XAS spectrum as shown in the main text (E_i = 706.5 and 707.5 eV). For comparison, the RIXS data taken away from these resonances are summarized in Supplementary Figure 3, where we compare the temperature evolution of RIXS spectra at the major post-edge region at E_i = 711 eV. The low-energy multiplet weight below 500 meV, specifically peak 1 ∼100 meV and peak 2 ∼220 meV that we assigned as spin-orbital excitations governed by trigonal lattice distortions, spin-orbit coupling and charge-transfer interactions (see main Figure 2), are much suppressed in intensity and are less defined in spectral structure compared to the RIXS data taken at E_i = 707.5 and 706.5 eV. Meanwhile, the relative weight of higher-energy fluorescence-like weight above 3-4 eV loss is enhanced compared to the E_i = 707.5 and 706.5 eV resonances. Both spectral components below 400 meV and above 3 eV exhibit weaker temperature evolution compared to E_i = 707.5 and 706.5 eV as well. This could be rationalized with enhanced contributions from ligand charge-transfer channels that are enabled at this excitation energy region, which likely account for the overall reduced sensitivity to spin and lattice nuances across the magnetic phase transition. This again highlights our main text data around the Fe L_3-edge XAS maximum that shows direct coupling to the fine structure of the multiplet dd transitions, such that the involved lattice distortions and spin-orbit interactions give rise to a strong fingerprint to the underlying magnetism.

Supplementary Note 3 Spectral fitting for RIXS data

To quantify the spectral weights of the low-energy excitations of interest below 500 meV loss, we applied a fitting model with pseudo-Voigt functions (1) to describe the spectral profile.

$$f(A,\mu,\sigma,\alpha) = \frac{(1-\alpha)A}{\sigma_g\sqrt{2\pi}}e^{-[(x-\mu)^2/2\sigma_g^2]} + \frac{\alpha A}{\pi}[\frac{\sigma_l}{(x-\mu)^2+(\sigma_l)^2}] \quad (1)$$

in which A is a fitting coefficient, and $\sigma_g = \sigma_l/\sqrt{2ln2}$. To fit the elastic line, a Lorentzian contribution $\alpha = 0.46$ is employed for the relative weight between Gaussian and Lorentzian contribution in the pseudo-Voigt function. The full widths at half maximum ($2\sigma_l$) of the elastic line at zero-energy loss, as well as the spin-orbital multiplets peak 1 (∼100 meV) and peak 2 (∼220 meV), are fixed to 90, 120, and 120 meV. These values are kept fixed after optimizing and relaxing the fitting constraints with the instrumental energy resolution of ≃85 meV, thus reducing the number of free parameters. Finite residual weight is inferred at the asymmetric higher-energy tail of peak 2, which is compensated by a resolution-limited component. The fitting examples at different temperature are shown in

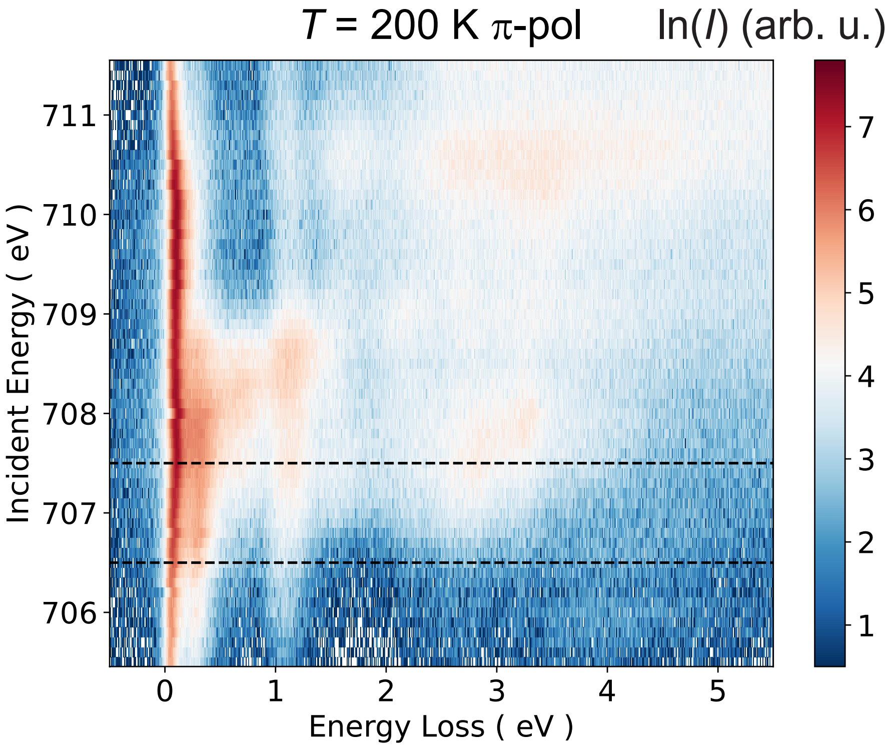

Supplementary Figure 2: Incident-energy dependent RIXS map taken at 200 K with π polarization. The RIXS intensity is plotted in logarithmic scale.

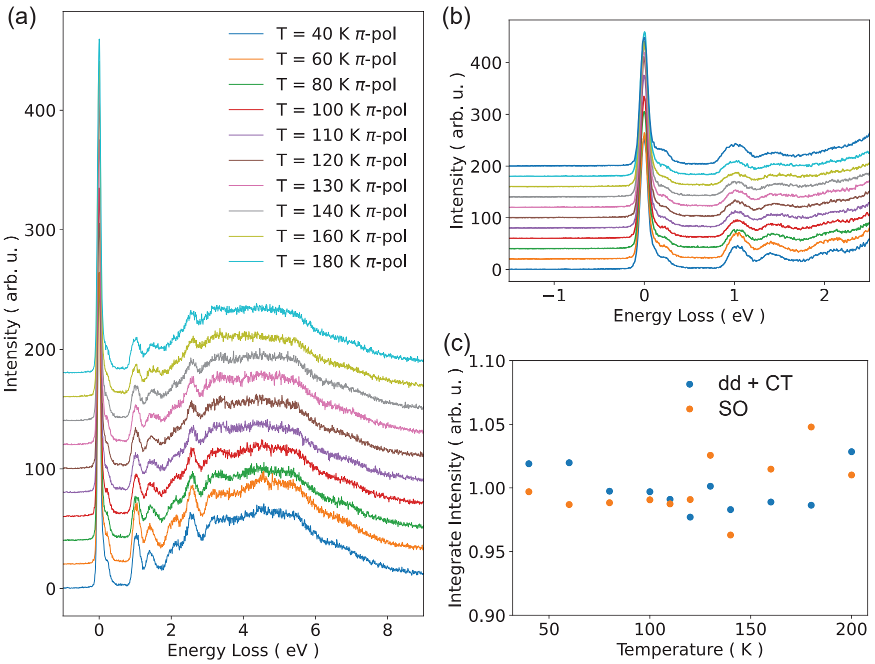

Supplementary Figure 3: RIXS data at $E_i = 711$ eV. a, A waterfall plot to present a comprehensive overview of the RIXS spectra at different temperatures. b, Zoom in on the low-energy regime. c, Integrated intensity of higher-energy dd and charge-transfer (CT) modes, as well as the low-energy spin-orbital (SO) excitations (covering peak 1 ∼100 eV and peak 2 ∼220 eV), respectively. The blue scatters represent the range of 3 to 6 eV for higher-energy dd and CT modes, and orange scatters represent the range of 0.1 to 0.4 eV for SO peaks 1 and 2. Intensity is normalized by an average factor for a clearer visualization of overall intensity evolution.

Supplementary Figure 4.

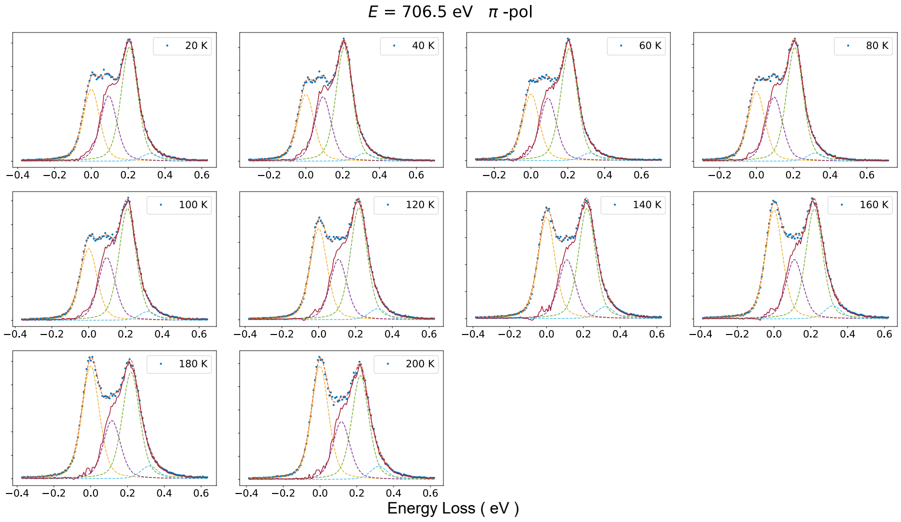

Supplementary Figure 4: Exemplary fitting scheme of RIXS spectra as a function of temperature. The raw data (blue filled markers), as well as spectral components of elastic line (orange dotted line), peak 1 ∼100 meV (purple dotted line), peak 2 ∼220 meV (green dotted line), and remaining higher-energy tail weight of peak 2 (cyan dotted line) are shown respectively. A elastic-subtracted version of spectral profile is also overlaid here (brown solid line).

Supplementary Note 4 Angle dependent RIXS data

In this section, we show incidence angle dependent RIXS results of the measured spin-orbital multiplet excitations and the elastic scattering at base temperature 20 K. In particular, we focus on the angular dependence of the spin-orbital excitations peak 1 and 2 in the antiferromagnetic state. In Supplementary Figure 5, the mode energies and spectral intensities of peak 1 and peak 2 are plotted for both π and σ incident light polarization. The spectral components are fitted by pseudo-Voigt functions as described in the main text and Supplementary Note 3. These measurements are carried out by varying the incident X-ray angle with respect to the sample surface while fixing the scattering angle 2θ. With this procedure, we infer that both peaks 1 and 2 exhibit a flat energy dispersion as compared to their broad spectral width. Some finite energy differences in these assigned multiplet manifolds with varied angles are due to RIXS cross-section changes with scattering geometry, which depends on the incident light angle and polarization [1]. Notably, here we express the changes by the correspond-

ing incident angle for referring to such scenario. On the other hand, the spectral intensities for peak 1 and 2 are plotted similarly with angle and compared with the elastic scattering. The overall sinusoidal-like trend for peak 1, peak 2 and elastic line agree with the combination of saturation and self-absorption effects in previous reports on multiplet excitations in Co^{3+} $3d^6$ compounds [2]. Namely, the scattered photons can get re-absorbed differently for varied incident angles, imposing spectral distortions on top of the RIXS cross-section. The actual absorption coefficients are nearly constants in our experimental configuration.

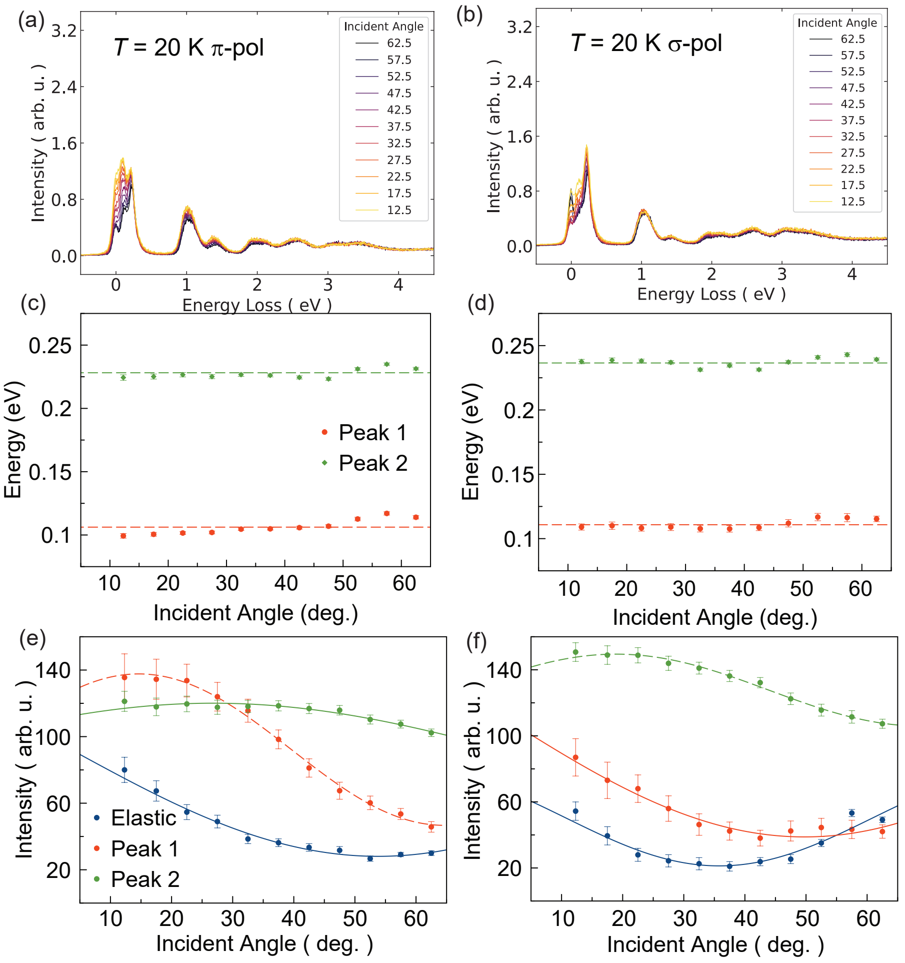

Supplementary Figure 5: Momentum dependent RIXS data for spin-orbital multiplet excitations peak 1 and 2. a,b Displaying raw data for different incident angles. c,d The fitted mode energies and e-f, spectral intensities are plotted as function of the incident angle of X-rays with respect to the sample surface.

Supplementary Note 5 Additional multiplet theory calculations

Here we provide additional details of the charge transfer multiplet (CTM) calculations. Particularly, we elaborate on the hypothesis of enhanced spin-singlet multiplet ground states in the paramagnetic phase.

5.1 XAS calculations

Supplementary Figure 6 shows the calculated Fe L-edge XAS of $FePS_3$ based on our parameter optimization from RIXS. The calculated spectra agree well with our measurements and with previous XAS measurements on $FePS_3$ [3].

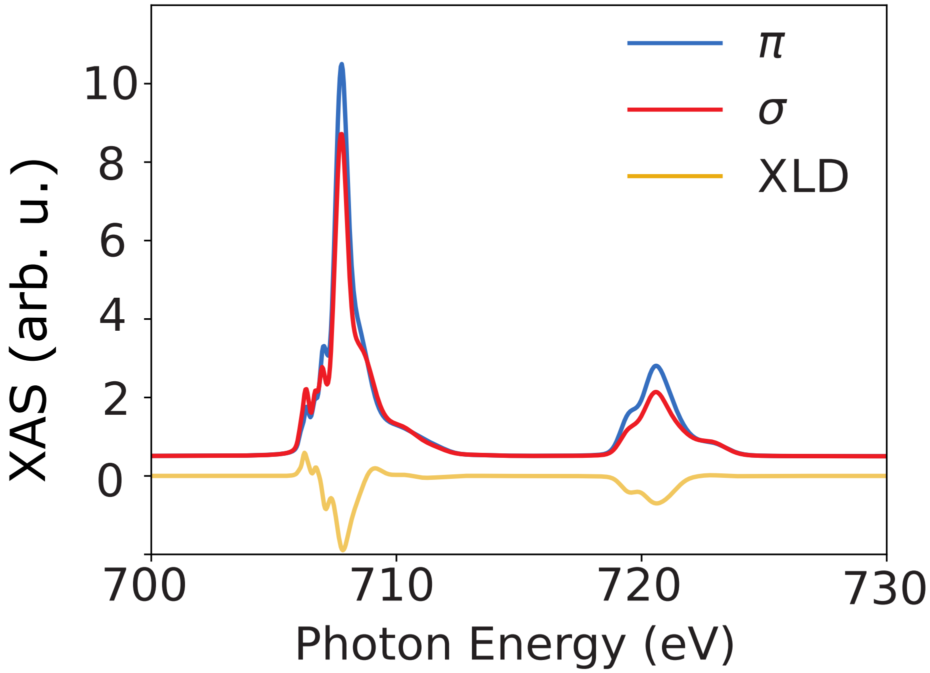

Supplementary Figure 6: Fe L-edge XAS calculations of $FePS_3$. The calculations are performed based on our parameter optimization from RIXS.

5.2 Hypothesized singlet $^5A_1$ ground state in paramagnetic state

In this section, we introduce a possible scenario to explain our observation of the temperature evolution with an enhanced zero-energy elastic peak when heating above the antiferromagnetic ordering temperature. We find that the

zero-energy elastic line exhibits increased intensity with a steep rise in vicinity to the antiferromagnetic transition ∼120 K. Here we postulate a mechanism based on the proximity and strong energy dispersion of the singlet ground state to explain this phenomenon. This has been observed in multiplet ground and excited state transitions of other magnetically-ordered systems, where the spin- and orbital-singlet components at zero-energy loss are enhanced with higher state symmetry in accordance to the magnetic (and lattice) structure in the high-temperature paramagnetic phase [4, 5].

Our calculations show that one of the main effects of charge transfer is that it lowers the energy of the $^1A_1$ state in comparison to the ionic calculations as seen in Supplementary Figure 8 in comparison to the energy level diagram presented in the manuscript. We propose that the shoulder observed in Supplementary Figure 6 corresponds to an excitation to $^1A_1$. However, we find that the $^1A_1$ strongly disperses as a function of distortion, and predict that it can mix with the ground state at high temperature as a result of the strong spin-phonon coupling in the system. Supplementary Figure 7 shows how a small D_τ distortion of ∼ 50 meV can lead to the stabilization of a $^1A_1$ ground state.

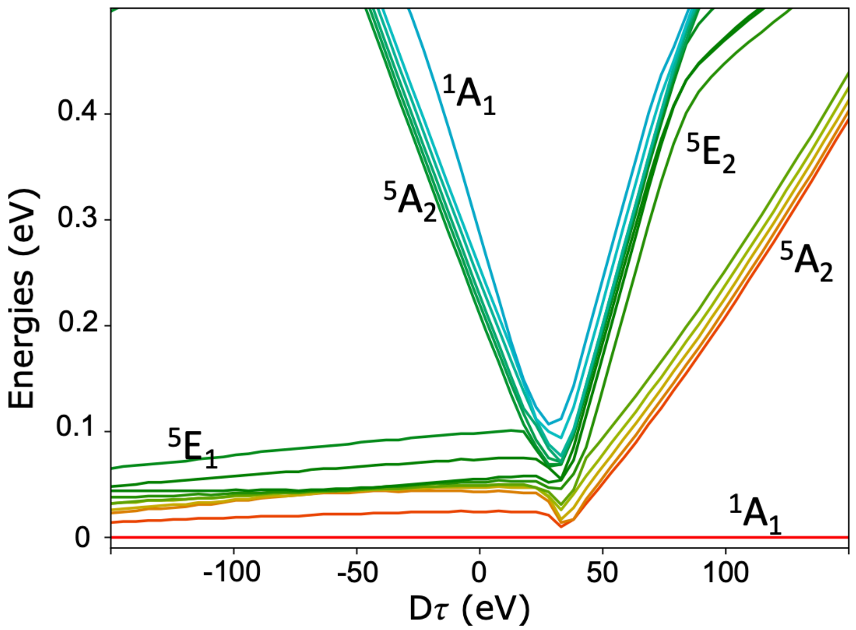

Supplementary Figure 7: Energy level diagram of Fe in $FePS_3$ as a function of D_τ with $10D_q$ = 1 eV and D_σ = -60 meV.

On the other hand, the RIXS elastic signal involves multiple other contributions that cannot be solely and predominantly contributed by the multiplet excitations, e.g. resolution-limited excitations such as magnons/phonons, diffuse

scattering from surface roughness, etc. Consequently, a thorough assessment of the zero-energy (quasi-) elastic scattering as such would require further investigations, as all these factors could account for systematic and experimental uncertainties.

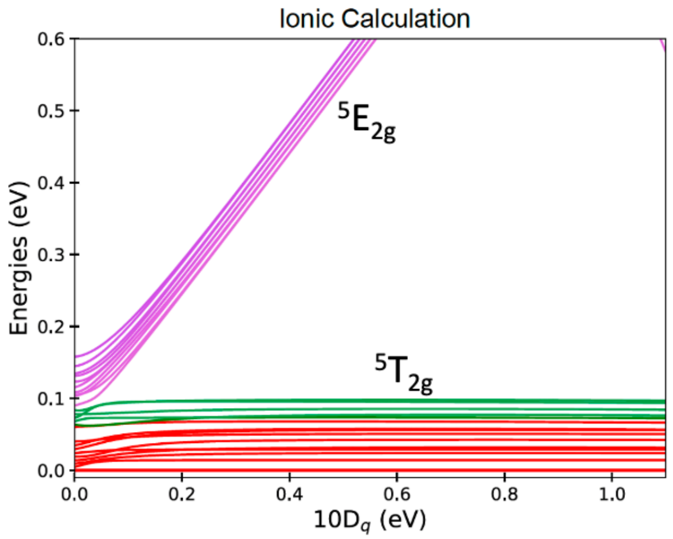

Supplementary Figure 8: Energy level diagram of Fe in $FePS_3$ as a function of distortion parameter $10D_q$. Here the calculation is ionic with no charge transfer included.

5.3 Effect of spin-orbit coupling

Supplementary Figure 9 shows the effect of spin-orbit coupling and magnetic exchange interaction on the energy levels of Fe in $FePS_3$ The energy splittings seen in Supplementary Figure 9a between the energy states of the $^5E_1$ ground state multiplet are due to the exchange interaction. When the spin-orbit coupling is not quenched, this splitting increases and the $^5E_1$ spans 100 meV as seen in Supplementary Figure 9b.

Supplementary Note 6 Temperature-dependent evolution of low-energy excitations

Supplementary Figure 10 illustrates the temperature dependence of the RIXS intensity for the low-energy excitations of peak 1 and 2. The data show that

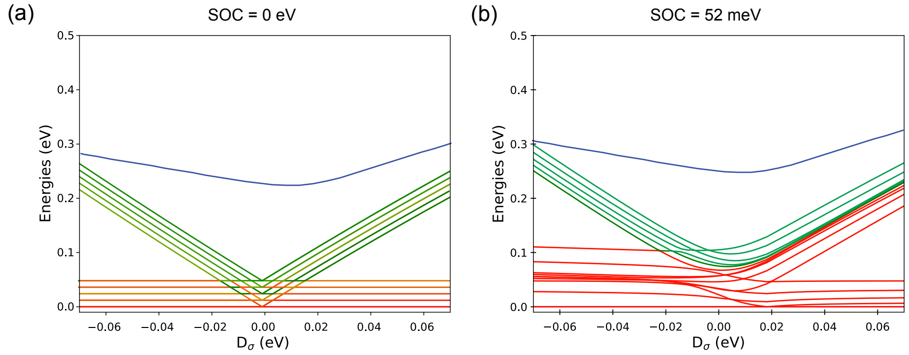

Supplementary Figure 9: Energy level diagram of Fe in $FePS_3$. a, Calculation done without spin-orbit coupling. b, with spin-orbit coupling set to its full atomic value of 52 meV.

both low-energy excitation peaks exhibit significant variation with temperature, suggesting an order-parameter evolution of these excitations. Meanwhile, the intensity of the elastic line shows a step like jump, indicating a more pronounced temperature-dependent change in this component.

Supplementary Note 7 Temperature dependent magnetic calculations

7.1 Magnetic susceptibility calculations

Here we show our mean-field self-consistent calculation of the magnetic susceptibility in Supplementary Figure 11a. Our model roughly predicts the Néel temperature and has a profile very close to the experimental susceptibility measurements [6] as shown in Supplementary Figure 11b. Although the profile is not not perfect, given that we approximate the exchange interaction with only one effective parameter in order to include it on a mean-field level in the RIXS calculation, the close resemblance validates our model. This mean-field magnetic-exchange interaction was used in the RIXS calculations as a function of temperature.

7.2 Alternative thermal development scenario for the spin-orbital multiplet excitations

Here we show calculations for the temperature dependence of the measured multiplet excitations in Supplementary Figure 12 without solving the mean-field model self-consistently and keeping the exchange interaction constant. This gives only a continuous trend displayed in Supplementary Figure 12 that is

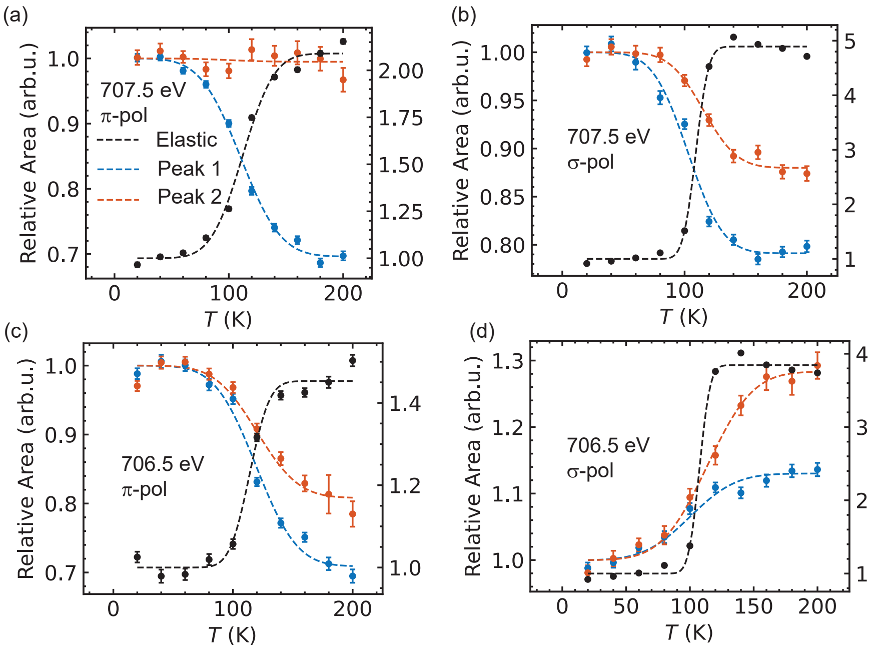

Supplementary Figure 10: Temperature dependence of RIXS Intensity for spin-orbital excitations and the elastic peak. The intensity values are normalized relative to the value at 20 K for comparative analysis. The intensity scales for the spin-orbital peaks and the elastic line are shown on the left and right axes, respectively. The dashed line is a guide to the eye.

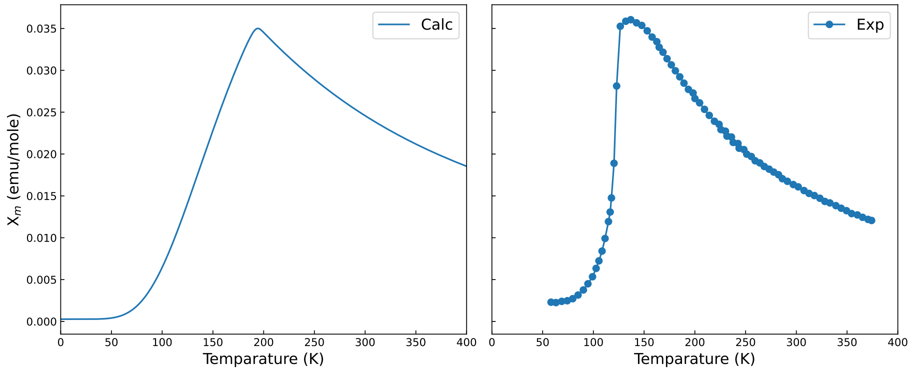

Supplementary Figure 11: Magnetic susceptibility of $FePS_3$. a, Calculated self-consistently using our mean-field model. b, Experimental measurement digitized from [6].

insensitive to the magnetic phase transitions, or other broadening factors if any.

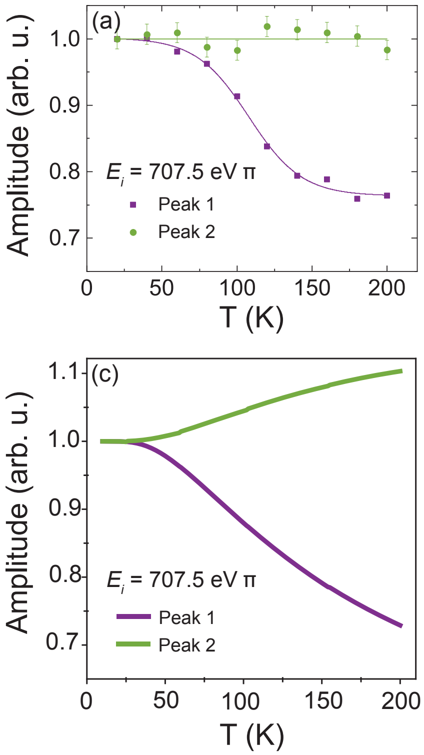

Supplementary Figure 12: Temperature dependence of the RIXS intensity of the spin-orbital excitations as we cross the magnetic transition temperature. a-b, Experimental results. c-d, Theoretical calculations where the exchange interaction is kept constant for all the temperatures.

Supplementary Note 8 Characterization of mechanically exfoliated flake samples

$FePS_3$ thin flake samples were meticulously prepared through 'scotch-tape' mechanical exfoliation of single crystalline materials. For the specific purpose of conducting X-ray measurements, isolated flake samples were meticulously crafted via van der Waals force transfer. The process entailed initially mechanically exfoliating the sample onto a silicon substrate, followed by the precise selection of flakes. Subsequently, these selected flakes were delicately transferred onto a stack comprising a polycarbonate film atop a polydimethylsiloxane (PDMS) stack. The polycarbonate film was later removed using a chloroform

solution. These exfoliated crystals exhibited remarkable stability when exposed to ambient air conditions, with no discernible alterations observed under optical microscopy over a period of at least one week. To achieve the desired mask pattern, the areas enclosed by the mask were meticulously etched using a precision needle tip. By employing atomic force microscopy measurements, we conducted a spatial-dependent survey over the exfoliated sample terraces, and evaluated the height distribution for assessing the flake thickness. This is shown in Supplementary Figure 13. The thickness of the monolayer is 0.78 nm [7], indicating that the flake is composed of 4-5 layers.

To measure the spatially separated flake samples, we employed an aperture horizontal slit at the exit slit position of the upstream beamline optics to further trim the X-ray beam focusing, achieving a footprint of $\sim 20 \times 4\ \mu m^2$ (horizontal×vertical). For the 50 ML and 5 ML flake samples, the X-ray measurements were performed with a scattering angle $2\theta = 130°$ for the experimental geometry. The scattering plane is fixed to the bc plane with the in-plane momentum transfer along the crystallographic [010] direction in the normal incidence configuration. σ polarization was employed for the incident X-rays.

Supplementary Note 9 Examination on beam damage effects

Supplementary Figure 14 shows the XAS and RIXS spectra when recorded at distinct course of acquisition time in our experiments. No clear signature of beam-induced temporal changes were observed from the spectral appearances.

References

[1] de Groot, F. M. *et al.* 2p X-ray absorption spectroscopy of 3D transition metal systems. *Journal of Electron Spectroscopy and Related Phenomena* **249**, 147061 (2021).

[2] Wang, R. P. *et al.* Saturation and self-absorption effects in the angle-dependent 2p3d resonant inelastic X-ray scattering spectra of Co3+. *Journal of Synchrotron Radiation* **27**, 979–987 (2020).

[3] Chang, A. G. *et al.* Trigonal distortion in zigzag-antiferromagnet iron phosphorus trisulfide. *Physical Review B* **106**, 125412 (2022).

[4] Miao, L. *et al.* High temperature singlet-based magnetism from Hund's rule correlations. *Nature Communications* **10**, 644 (2019).

[5] Marino, A. *et al.* Singlet magnetism in intermetallic UGa2 unveiled by inelastic x-ray scattering. *Physical Review B* **108**, 045142 (2023).

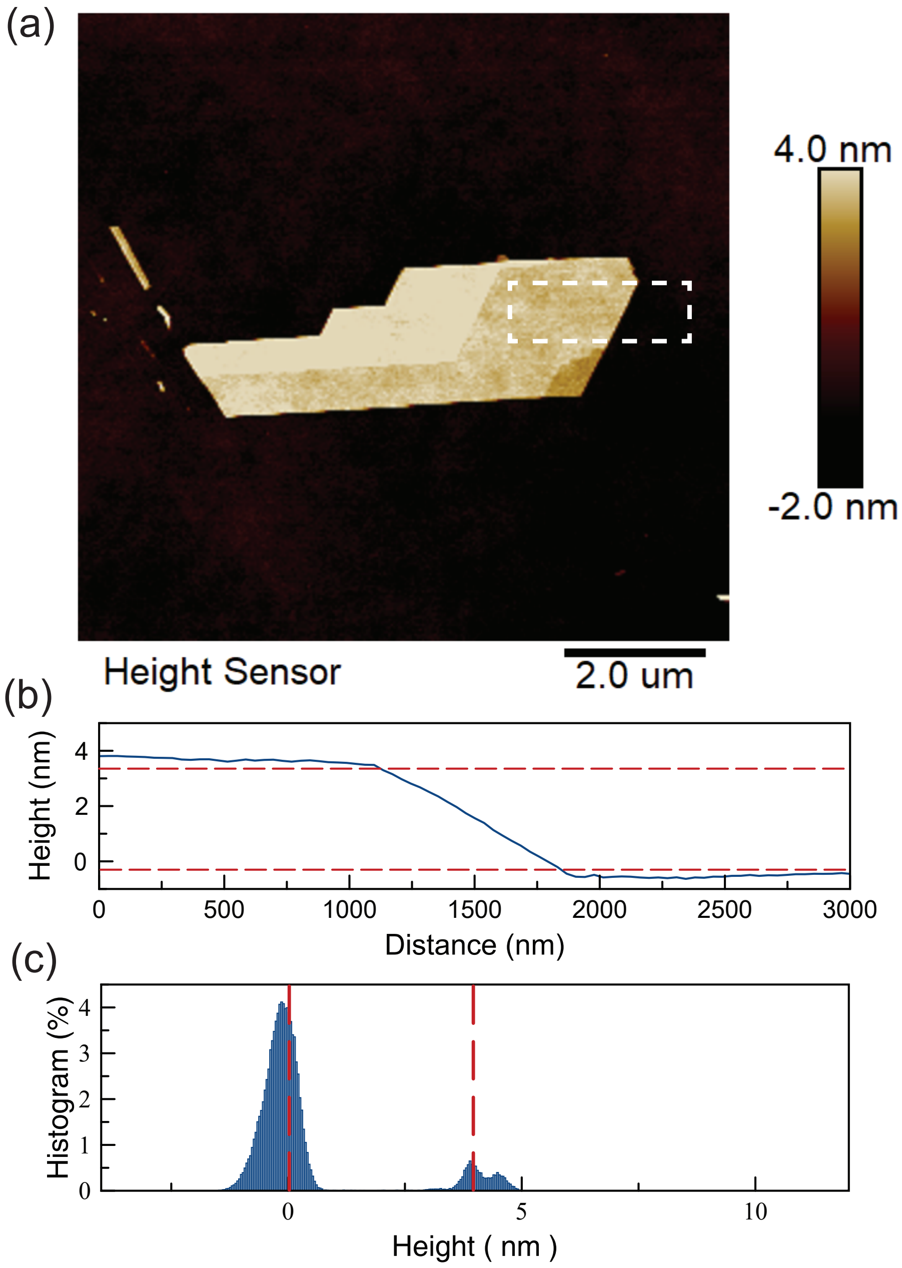

Supplementary Figure 13: a, Atomic force microscopy image of exfoliated $FePS_3$ samples. The white dash box indicates the height measurement area. b, The height difference in the direction across the edge. The red dotted line is the visual extension of the height step. c, The histogram of the heights in the scanning area, the red dash lines are guides to the eyes.

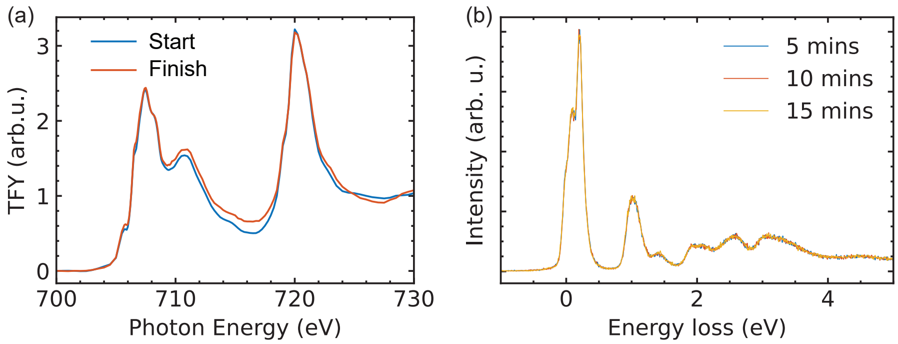

Supplementary Figure 14: a, XAS spectral comparison before/after a one-week synchrotron beamtime experiment. b, RIXS spectral comparison when recorded over an acquisition time of 5, 10, and 15 minutes, respectively.

[6] Joy, P. A. & Vasudevan, S. Magnetism in the layered transition-metal thiophosphates MPS3 (M = Mn, Fe, and Ni). *Physical Review B* **46**, 5425–5433 (1992).

[7] Wang, X. *et al.* Raman spectroscopy of atomically thin two-dimensional magnetic iron phosphorus trisulfide (FePS3) crystals. *2D Materials* **3**, 031009 (2016). URL `http://dx.doi.org/10.1088/2053-1583/3/3/031009`.
